# Novel Biomarker Candidates for Febrile Neutropenia in Hematological Patients Using Nontargeted Metabolomics

**DOI:** 10.1155/2018/6964529

**Published:** 2018-04-12

**Authors:** Marika Lappalainen, Jenna Jokkala, Auni Juutilainen, Sari Hämäläinen, Irma Koivula, Esa Jantunen, Kati Hanhineva, Kari Pulkki

**Affiliations:** ^1^Department of Internal Medicine, Central Hospital of Central Finland, Jyväskylä, Finland; ^2^Institute of Clinical Medicine/Internal Medicine, University of Eastern Finland, Kuopio, Finland; ^3^Institute of Public Health and Clinical Nutrition, University of Eastern Finland, Kuopio, Finland; ^4^Department of Medicine, Kuopio University Hospital, Kuopio, Finland; ^5^Siun Sote-Hospital District of North Carelia, Joensuu, Finland; ^6^Eastern Finland Laboratory Centre, Kuopio, Finland; ^7^Laboratory Division, Turku University Hospital, Turku, Finland; ^8^Clinical Chemistry, Faculty of Medicine, University of Turku, Turku, Finland

## Abstract

**Background:**

Novel potential small molecular biomarkers for sepsis were analyzed with nontargeted metabolite profiling to find biomarkers for febrile neutropenia after intensive chemotherapy for hematological malignancies.

**Methods:**

Altogether, 85 patients were included into this prospective study at the start of febrile neutropenia after intensive chemotherapy for acute myeloid leukemia or after autologous stem cell transplantation. The plasma samples for the nontargeted metabolite profiling analysis by liquid chromatography-mass spectrometry were taken when fever rose over 38° and on the next morning.

**Results:**

Altogether, 90 differential molecular features were shown to explain the differences between patients with complicated (bacteremia, severe sepsis, or fatal outcome) and noncomplicated courses of febrile neutropenia. The most differential compounds were an androgen hormone, citrulline, and phosphatidylethanolamine PE(18:0/20:4). The clinical relevance of the findings was evaluated by comparing them with conventional biomarkers like C-reactive protein and procalcitonin.

**Conclusion:**

These results hold promise to find out novel biomarkers for febrile neutropenia, including citrulline. Furthermore, androgen metabolism merits further studies.

## 1. Introduction

Febrile neutropenia is a common complication in hematological patients receiving intensive chemotherapy. Although a minority of these patients develop septic shock [1, 2], sepsis is still a major cause of morbidity and mortality during the neutropenic phase [3, 4]. In these patients, life-threatening complications can develop in hours depending on the pathogen. Not only C-reactive protein (CRP) and procalcitonin (PCT) but also several other biomarkers have been explored to identify patients at risk for complicated course of febrile neutropenia [5]. CRP and PCT both have some limitations such as nonspecificity and delayed response. PCT is superior to CRP for predictive purposes and is slightly more pathogen-dependent, especially in gram-negative bacteremia [3, 6]. However, there still is a need for more rapid and accurate markers, which also could be used for de-escalation strategies of broad-spectrum antibiotics.

Nontargeted metabolite profiling or metabolomics is a hypothesis-free study approach that focuses on finding differences in metabolite profiles between study subjects contributing to the discovery of novel small-sized molecular biomarkers for disease progression or prevention [7, 8]. In previous studies, metabolomics has been used to differentiate sepsis from systemic inflammatory response syndrome [9–12] and to detect prognostic biomarkers for septic shock [11, 13–15]. There is only one previous study including metabolomics in patients with febrile neutropenia. Richter et al. [16] found twenty-one biomarker candidates including three peptides, six proteins, and six phosphatidylcholines (PC), which identified febrile neutropenic patients with proven infection from those without it.

The use of metabolomics as a tool for discovery of diagnostic markers in febrile neutropenia is a novel approach. The purpose of our study was to evaluate the differences found in a metabolic profile and to identify potentially useful biomarker candidates for further validation to recognize early increased risk of adverse outcome in hematological patients with febrile neutropenia after intensive chemotherapy.

## 2. Patients and Methods

### 2.1. Patients

Between December 2009 and November 2012, altogether 85 hematological patients treated at the adult hematology ward of the Department of Medicine, Kuopio University Hospital, and who gave written permission were included into this prospective study. The inclusion criteria were fulfilled if the patient was ≤70 years old, if the patient received intensive chemotherapy for acute myeloid leukemia (AML), or if the patient was an autologous stem cell transplant (ASCT) recipient and had febrile neutropenia (see definitions later). There were 54 males and 31 females with a median age of 61 years (18–70 years). Twenty-three patients had AML, and 62 were ASCT recipients (40 non-Hodgkin lymphoma, 19 multiple myeloma, and 3 Hodgkin lymphoma). Only the first induction course of AML patients was included in the study.

All patients were carefully followed up at the hematology ward from the start of febrile neutropenia until recovery of neutropenia. Blood pressure, oxygen saturation, respiratory frequency, heart rate, skin temperature, urine output, and fluid intake were closely followed up. Each patient was examined daily thoroughly for clinical signs and sources of infections. Serum and plasma samples for laboratory analyses were taken at the onset of neutropenic fever on day 0 (d0) and further samples on the next morning (d1). Broad-spectrum antibiotics were started as soon as the samples for blood cultures had been taken. Fifty-four patients (64%) received granulocyte colony-stimulating factor to shorten the length of the neutropenic period.

The study setting was to investigate differences in the responses of patients with and without complicated course of febrile neutropenia on two consecutive days. The noncomplicated patient group was regarded as a control group.

### 2.2. Data Collection

Clinical data, including the hour and date of the start of fever, possible sites of infections, and hemodynamic parameters suggesting development of septic shock were recorded on a structured data collection form. Laboratory findings, including microbiological blood culture results, were registered.

### 2.3. Definitions

Febrile neutropenia was defined using the criteria from IDSA (Infectious Diseases Society of America) [17]. Neutropenia was defined as a neutrophil count < 0.5 × 10^9^/L or with a predicted decrease to <0.5 × 10^9^/L during the next 48 h. Fever was defined as a single oral temperature of ≥38.3°C or a temperature of ≥38.0°C sustained over a 1-hour period.

Sepsis was defined as a syndrome in which systemic inflammatory response was present with infection. Severe sepsis was defined as sepsis with organ dysfunction. Septic shock was defined if hypoperfusion (systolic arterial pressure < 90 mmHg, a mean arterial pressure < 60 mmHg, or a reduction in systolic blood pressure of >40 mmHg from baseline) was present despite adequate volume resuscitation, in the absence of other causes of hypotension [18, 19].

Complicated course of febrile neutropenia was defined as a positive blood culture finding and/or development of severe sepsis or septic shock during the period from the onset of febrile neutropenia until the recovery of neutropenia.

### 2.4. Laboratory Measurements

The concentration of serum CRP was measured with a Konelab 60i Clinical Chemistry Analyzer (Lab systems CLD, Konelab, Helsinki, Finland) or Cobas 6000 analyzer (Hitachi, Tokyo, Japan). The intra- and interassay CV% were 2.3–4.3%. The upper reference limit of serum or plasma CRP of a healthy reference population is 3 mg/L. Plasma PCT was measured from EDTA plasma using a Cobas 6000 analyzer (Hitachi, Tokyo, Japan) with a sensitivity of 0.06 *μ*g/L. The CVs (intra- and interassay) were 1.4% and 3.0% for 0.46 *μ*g/L and 1.1% and 2.6% for 9.4 *μ*g/L PCT, respectively. Blood cultures (2-3 sets including two bottles/set) were drawn immediately at the beginning of neutropenic fever (day 0), and an additional sampling was done if fever persisted for 3–5 days. They were processed using the automated blood culture system Bactec 9240 (Becton Dickinson, Sparks, MD, USA). The incubation episode for aerobic and anaerobic bottles was 7 days and for MYCO F/Lytic bottles 42 days. The plasma samples for metabolomics assays were stored frozen at −80°C until analyzed.

### 2.5. Nontargeted LC-MS Metabolite Profiling Analyses

The methods including sample preparation and analysis were similar as described previously [20]. In detail, the samples were prepared in 96-well plates by mixing an aliquot of 100 *μ*L of EDTA plasma samples with 400 *μ*L of acetonitrile (VWR International), mixed on a vortex at maximum speed for 15 s, incubated on an ice bath for 15 min to precipitate proteins, and centrifuged at 16,000 ×g for 10 min to filter the samples (0.2 *μ*m polytetrafluoroethylene filters in a 96-well plate) and collect the supernatant. Aliquots of 2 *μ*L were taken from at least half of the plasma samples, mixed together in a tube, and used as the quality control (QC) sample in the analysis; a solvent blank was prepared in the same manner.

The samples were analyzed by nontargeted liquid chromatography quadrupole time-of-flight mass spectrometry (LC-QTOF-MS) using the UHPLC-QTOF-MS system (Agilent Technologies, Karlsruhe, Germany), which consisted of a 1290 LC system, Jet Stream electrospray ionization (ESI), and 6540 UHD accurate-mass QTOF spectrometry. The samples were analyzed by using two different chromatographic techniques: reversed phase (RP) and hydrophilic interaction (HILIC) liquid chromatography. The sample tray was kept at 4°C during the analysis. The data acquisition software was the MassHunter Acquisition B.04.00 (Agilent Technologies). The QC samples were injected after every 12 samples and 10 samples at the beginning of the analysis. The order of the analysis of the samples was random.

In the RP technique, 2 *μ*L of the sample solution was injected onto the column (Zorbax Eclipse XDB-C18 Rapid-Resolution HD 1.8 *μ*m, 2.1 × 100 mm; Agilent Technologies, Palo Alto, CA, USA) and maintained at 50°C. The mobile phases, delivered at 0.4 mL/min, consisted of water (eluent A, Milli-Q purified; Millipore) and methanol (eluent B; Sigma-Aldrich), both containing 0.1% (*v*/*v*) of formic acid (Sigma-Aldrich). The following gradient profile was used: 2% → 100% B (0–10 min), 100% B (10–14.50 min), 100% → 2%B (14.50–14.51 min), and 2% B (14.51–16.50 min).

In the HILIC technique, 2 *μ*L of the sample solution was injected onto the column (Acquity UPLC BEH Amide 1.7 *μ*m, 2.1 × 100 mm, Waters Corporation, Milford, MA, USA) and maintained at 45°C. The mobile phases, delivered at 0.6 mL/min, consisted of 50% acetonitrile in water (*v*/*v*; eluent A) and 90% acetonitrile in water (*v*/*v*; eluent B), both containing 20 mmol/L ammonium formate, pH 3 (Sigma-Aldrich). The following gradient profile was used: 100% B (0–2.5 min), 100% → 0% B (2.5–10 min), 0% → 100% B (10.0–10.01 min), and 100% B (10.01–12.50 min).

The MS conditions after both chromatographic analyses were as follows: Jet Stream ESI source, operated in positive and negative ionization mode, a drying gas temperature 325°C, gas flow 10 L/min, a sheath gas temperature 350°C, sheath gas flow 11 L/min, nebulizer pressure 45 pounds per square inch, capillary voltage 3500 V, nozzle voltage 1000 V, fragmentor voltage 100 V, and a skimmer 45 V. For data acquisition, a 2 GHz extended dynamic range mode was used, and the instrument was set to acquire over the *m*/*z* 20–1600. Data were collected in the centroid mode at the acquisition rate of 1.67 spectra/s (i.e., 600 ms/spectrum) with an abundance threshold of 150. For the automatic data-dependent MS/MS analyses performed on the QC samples, the 4 most abundant ions were selected for fragmentation from every precursor scan cycle with a scan rate 3.33 spectra/s. These ions were excluded after two product ion spectra and released again for fragmentation after a 0.25 min hold. The precursor scan time was based on ion intensity, ending at 20,000 counts or after 200 ms in HILIC and 25,000 counts or 200 ms in RP, respectively. The product ion scan time was 200 ms. Collision energies used were 10, 20, and 40 V in subsequent assays. The continuous mass axis calibration was performed monitoring two reference ions from an infusion solution throughout the assays: *m*/*z* 121.05087300 and 922.00979800 in positive mode and *m*/*z* 112.98558700 and 966.00072500 in negative mode.

### 2.6. Metabolomics Data Analysis

Liquid chromatography-mass spectrometry (LC-MS) data were collected first with the “Find by Molecular Feature” algorithm (MassHunter Qualitative Analysis B.06.00, Agilent Technologies, USA). The peak collection threshold was 1000–4000 counts depending on chromatography mode, and the allowed ion species were restricted to [M+H]+ in ESI(+) and [M−H]− in ESI(−). Data files (.cef format) were exported to Mass Profiler Professional version 14.0 (Agilent Technologies) for peak alignment to create a list of potential molecular features. The molecular features were restricted to those present at least in 50% of samples within one study group, and the resulting entity list was used for feature-specific data reanalyzation back from raw data with the “Find by Formula” algorithm (MassHunter Qualitative Analysis B.06.00). For this recursive analysis, compound mass tolerance was ±15.00 ppm, retention time ± 0.1 min, and symmetric expansion value for chromatograms ± 35.0 ppm. The resulting peak data was again aligned with Mass Profiler Professional software and cleaned by filtering (metabolite features that were present in at least 80% of samples in any of the four study groups) resulting in 417, 420, 2385, and 1276 molecular features from HILIC ESI(+), HILIC ESI(−), RP ESI(+), and RP ESI(−), respectively.

The metabolite features were further subjected for statistical analysis by a pair-wise comparison of the case group consisting of patients with complicated course of febrile neutropenia and the control group consisting of noncomplicated febrile neutropenia either at day 0 or day 1 by Student's *t*-test. The resulting *p* values were adjusted for multiple comparisons by Benjamini-Hochberg false discovery rate (FDR) correction within each of the four analytical approaches [21]. Finally, the four datasets were exported into Microsoft Excel.

The data from each of the four analytical approaches were subjected to unsupervised principal component analysis (PCA) and supervised classification algorithm partial least-squares discriminant analysis (PLS-DA; SIMCA 14, Umetrics, Sweden). The data were log10-transformed and Pareto-scaled, and the model was validated by SIMCA 13 internal cross validation [22, 23]. PLS-DA illustrates the differences between the two study groups at either day 0 or day 1, separately, and gives variable importance projection (VIP) values: the larger the VIP value is, the more significant contributor the metabolite is in the model. The resulting VIP values for each metabolite were integrated in the data and used for classifying out the most important metabolite features. Due to small group size and high variability in the group, the cut-off VIP value was set to 1.5.

The metabolite features were further filtered according to an average peak area > 50,000 and molecular mass < 1000 Da to exclude small and insignificant features from the analysis resulting in a set of 1935 molecular features (144 HILIC ESI(−), 152 HILIC ESI(+), 517 RP ESI(−), and 1122 RP ESI(+)). The molecular features with a VIP value on day 0 and day 1 > 1.5 and the corrected *p* value < 0.05 on either day 0 or day 1 were considered the most significant markers. Molecular features with a VIP value > 1.5 on day 0 and day 1 but with a noncorrected *p* value < 0.05 also on both days were considered as the second most important molecular features. Also, molecular features with a VIP value > 1.5 either on day 0 or on day 1 and a *p* value < 0.05 on day 0 and day 1 were considered important and subjected for identification.

The identification of metabolites was based on MS/MS fragmentation spectra acquired in the automatic MS/MS analysis during the initial data acquisition or later on via reinjection of the samples. The spectra were compared with the in-house standard-compound library, METLIN Metabolomics Database [24], the Human Metabolome Database [25], and LIPID MAPS [26] or fragmentation patterns characteristic for certain metabolite types including phospholipids [27, 28]. Shortly, the identification of PCs was based on the presence of a protonated head group (*m*/*z* 184.073) in positive ESI mode and presence of formic acid adduct [M+COO]−, neutral loss of M+COO−60.0182, and the size of fatty acyl side-chain fragments in the negative ESI mode spectra. Phosphatidylethanolamines (PE) were identified based on the characteristic neutral loss of 141.027 Da in the positive ESI mode and fatty acyl side-chain fragments in the negative ESI mode spectra. The identification of androsterone/5*α*-dihydrotestosterone sulfate (ADTS/DHTS) was based on sulfate fragment SO_4_H_2_ of 96.9591 Da and exact molecular mass 370.1815 Da. However, as the sulfate fragment is highly dominant and the fragmentation pattern shows no other clear fragments, the metabolite was annotated as ADTS/DHTS. MS/MS fragmentation data for all the identified metabolites is presented in Table 1.

### 2.7. The Statistical Analysis of the Conventional Biomarkers

CRP and PCT values were expressed as medians and interquartiles due to the skewed distribution. The Mann–Whitney *U* test was used to detect differences between the groups in continuous variables. The association between categorical variables was studied by *χ* ^2^ test with Spearman's correlation. A *p* value less than 0.05 was considered significant. Data analyses were conducted with SPSS 21.0 Software (SPSS Inc., Chicago, Illinois, USA).

## 3. Results

### 3.1. Course of Febrile Neutropenia and Blood Culture Findings

The characteristics of patients with complicated course of febrile neutropenia are presented in Table 2. Altogether, the group included twenty patients (24%). Twelve patients had bacteremia without any other signs of complication. Eight patients fulfilled the criteria for complicated sepsis, and three of them developed septic shock. Altogether, six patients needed intensive care unit treatment and three of them died (mortality 3% in the whole series).

The blood cultures were positive in 18 out of 85 patients (21%) with fourteen gram-positive and three gram-negative bacteremias, respectively. The gram-positive findings included *Enterococcus faecium* (*n* = 5), *Staphylococcus epidermidis* (*n* = 3), *Streptococcus mitis* (*n* = 2), *Staphylococcus haemolyticus* (*n* = 2), *Streptococcus salivarius* (*n* = 1), and *Gemella morbillorum* (*n* = 1). The gram-negative findings were *Klebsiella oxytoca* (*n* = 1), *Escherichia coli* (*n* = 1), and *Pseudomonas aeruginosa* (*n* = 1). One case of fungemia was found (*Candida krusei*).

### 3.2. Conventional Biomarkers

The medians (IQ) of CRP for patients with complicated course of febrile neutropenia were 51 (26–100) on d0 and 100 (57–214) on d1. In patients without complications, CRP values were 36 (19–67) on d0 and 67 (35–95) on d1. There was a significant statistical difference on d1 (*p* = 0.014). The medians (IQ) of PCT for patients with complicated course of febrile neutropenia were 0.164 (0.120–0.279) on d0 and 0.350 (0.190–1.855) on d1. In patients without complications, PCT values were 0.122 (0.077–0.193) on d0 and 0.163 (0.104–0.320) on d1. There was a significant statistical difference between the groups on both days. *p* values were 0.027 and 0.020 on day d0 and d1, respectively.

### 3.3. Nontargeted Metabolite Profiling

**Figure 1:**
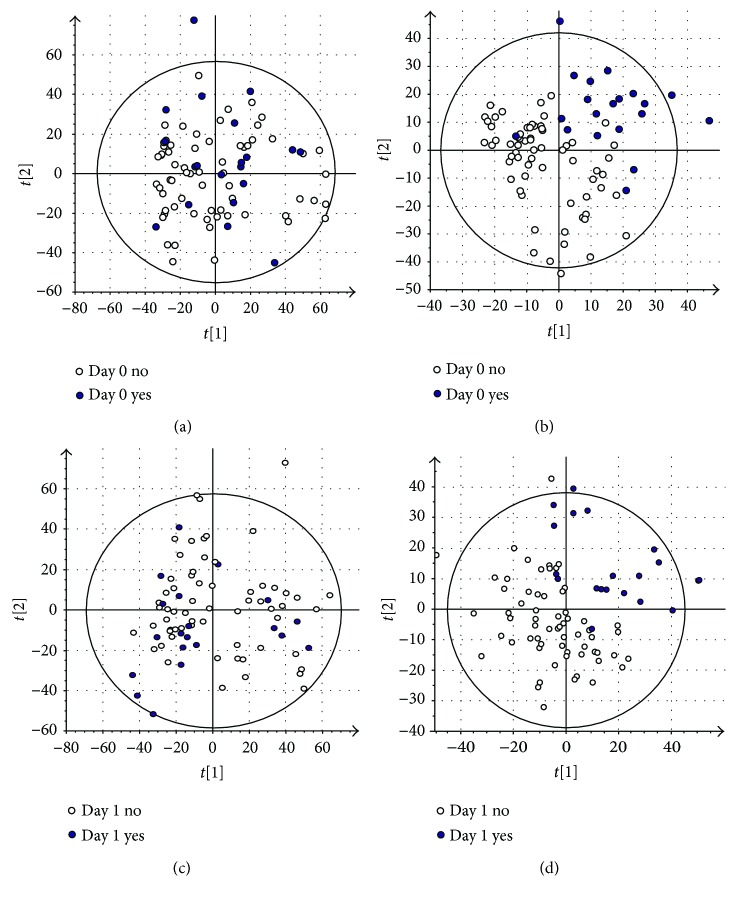
Panels (a) and (c) show the first two components of principal component analysis (PCA) for all the molecular features (log-transformed) from day 0 and day 1, respectively. Panels (b) and (d) show the first two components of a partial least-squares discriminant analysis (PLS-DA) for all the molecular features (log-transformed) from day 0 and day 1, respectively. In both PLS-DA models ((b) and (d)), six components cumulatively explain 97% of the variance in the data between the groups on day 0 (b) and on day 1 (d). These six components explain 49% and 58% of the variance in the data on day 0 and day 1, respectively. White dots are patients without complicated course of febrile neutropenia. Black dots are patients with complicated course of febrile neutropenia.

Principal component analysis of the molecular features collected at four analytical modes of LC-MS analysis is presented in the Supplementary Figure (available here). Altogether, 90 molecular features fulfilled the filtering criteria and were considered to be important for explaining the metabolic differences between patients with noncomplicated versus complicated course of febrile neutropenia. Of these 90 molecular features, 52 were tentatively identified corresponding to 25 different metabolites (Table 1) (Figure 1).

**Figure 2:**
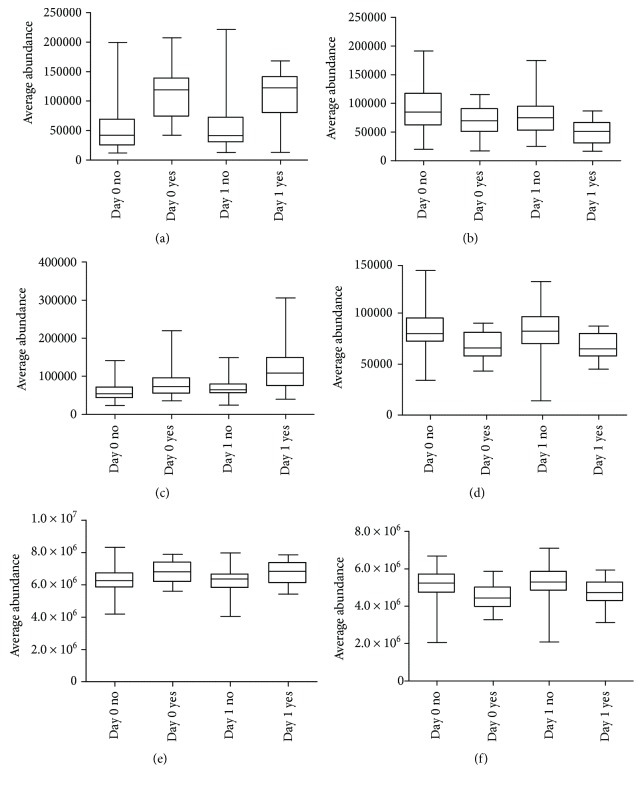
The most differential metabolites and their changes on day 0 (d0) and day 1 (d1) from beginning of the febrile neutropenia. (a) Androsterone sulfate (ADTS)/dihydrotestosterone sulfate (DHTS), (b) citrulline, (c) phosphatidylethanolamine PE(18:0/20:4), (d) phosphatidylcholine PC(15:0/22:6), (e) phosphatidylcholine PC(16:0/20:4), and (f) phosphatidylcholine PC(16:0/22:6). “No” indicates patients without complicated course of febrile neutropenia. “Yes” indicates patients with complicated course of febrile neutropenia. Day 0 indicates the onset of febrile neutropenia. Day 1 indicates the next day. Horizontal line represents median, box encompasses 25th and 75th percentiles, and error bar encompasses 10th and 90th percentiles.

Six molecular features fulfilled the strictest filtering criteria and were considered as the most significant markers to differentiate between noncomplicated and complicated course of febrile neutropenia (Figure 2). Of these markers, one metabolite was unambiguously identified to be citrulline and two others tentatively identified to be ADTS/DHTS and PE(18:0/20:4), whereas three remained unidentified (Table 1). While ADTS/DHTS and PE(18:0/20:4) were significantly increased, citrulline demonstrated low levels in patients with complicated course of febrile neutropenia (Figure 2).

Twelve molecular features fulfilled the second most strict criteria and were considered important in explaining the differences. These features were tentatively identified to correspond to four different lipid metabolites, namely, PC(15:0/22:6), PC(16:0/20:4), PC(16:0/22:6), and PE(16:0/22:6) (Table 1). While PC with 20 : 4 fatty-acyl side chain increased, the PCs with 22 : 6 side chain decreased in patients with complicated course of febrile neutropenia (Figure 2). PE(16:0/22:6) showed a similar increase in patients with complicated course of febrile neutropenia as PE(18:0/20:4), but it did not meet the criteria set for the most significant markers due to a large relative standard deviation (Table 1).

Additionally, 19 metabolites were identified which fulfilled the lowest criteria. These tentatively identified metabolites containing various lysophosphatidylcholines and phosphatidylcholines, butyryl-L-carnitine, L-histidine, and putative pregnenolone sulfate (Table 1). All of the LysoPCs showed decreased levels in patients with complicated course of febrile neutropenia. Whereas in the case of PCs, a similar behavior was seen as with more important metabolite markers as the PCs containing a 22 : 6 fatty-acyl side chain, as they showed decreased levels in patients with complicated course of febrile neutropenia, while other PCs were increased in the complicated group. Butyryl-L-carnitine and tentative pregnenolone sulfate showed increased levels and L-histidine decreased levels in patients with complicated course of febrile neutropenia (Table 1).

### 3.4. Correlation of the Metabolomics Findings with Conventional Biomarkers

ADTS/DHTS had significant correlations with CRP and PCT on both days. With CRP, the *p* values were 0.002 and 0.003 on d0 and d1, respectively, and with PCT they were <0.001 on both days. Citrulline had a significant correlation on d1 with CRP (*p* < 0.001) and PCT (*p* = 0.001). PE(18:0/22:4) had a significant correlation with PCT on both time points (*p* = 0.016 and <0.001 on d0 and d1, resp.), but with CRP only on d1 (*p* = 0.005). Naturally, ADTS/DHTS had a significant difference according to gender (Table 3). However, when looking at the differences only among men, the significant difference remained between groups of complicated versus noncomplicated course of febrile neutropenia (*p* values < 0.001 and 0.001 on days d0 and d1, resp.). Also, women with complicated course of febrile neutropenia had higher ADTS/DHTS values than women without complications on d0 had (*p* = 0.045) (Table 4). The effects of andropause and menopause were evaluated by dividing patients to those under fifty years of age and those over fifty years (Table 5). Citrulline or PE(18:0/22:4) levels did not have any difference due to gender or age (data not shown).

## 4. Discussion

In this study, we describe how plasma samples from patients with hematological malignancy were analyzed with nontargeted metabolite profiling to find out potential novel small molecular biomarker candidates for complicated course of febrile neutropenia. This study is until now the largest study evaluating metabolite profiles in hematological patients with febrile neutropenia after intensive chemotherapy. ADTS/DHTS, citrulline, and PE(18:0/22:4) were all found to be important metabolic features differentiating patients with complicated versus noncomplicated course of febrile neutropenia at the start of fever (d0 and d1). These metabolites need further studies and validation in this patient cohort, where early and predictive biomarkers are urgently needed.

**Figure 3:**
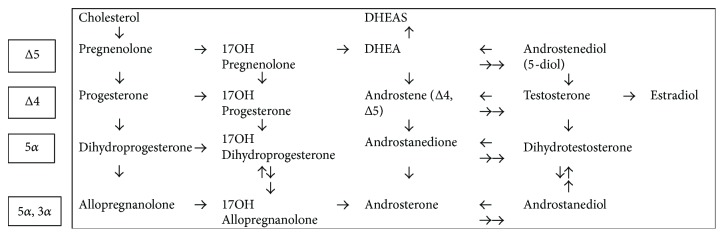
Schematic representation for the metabolite route of testosterone and dihydrotestosterone. Front door pathway (∆4 and ∆5 via testosterone) and the backdoor pathway (5*α* and 5*α*, 3*α* routes not via testosterone). Modified from Fukami et al. [32] and Luu-The [31].

The most significant molecular feature was tentatively identified to be either ADTS or DHTS, which increased significantly in patients with complicated course of febrile neutropenia. ADT and its sulfonated form androsterone sulfate (ADTS) are intermediates in the metabolic route of testosterone and its metabolite, dihydrotestosterone (DHT) [29]. The conversion of dehydroepiandrosterone DHEA to potent androgens and/or estrogens takes place in peripheral tissues by the 17*β*-HSD enzyme family [30]. In the testis and in the muscle, the conversion occurs via testosterone to DHT through the conventional front door pathway where DHEA is transformed to 4-androstenedione (4-dione) and further converted into testosterone. In tissues that express 5*α*-reductase, such as the prostate, liver, and skin, DHT is produced mostly from 4-dione without testosterone by the backdoor pathway (Figure 3) [31, 32]. Also, monocyte-derived macrophages have the ability to convert DHEAS to androgens [33].

To the best of our knowledge, there are no previous studies of ADT or ADTS in sepsis patients. Androstanedione is the intermediate between ADT and DHT in the backdoor pathway, and it has been studied as a part of adrenal and testicular steroidogenesis in patients with burn trauma and in intensive care unit patients, but findings have been contradictory [34–36]. In animal and *in vitro* studies, immunomodulatory effects of testosterone on macrophage function are mediated via the 5*α*-reductase-dependent conversion of testosterone to DHT [37, 38].

In patients with sepsis, testosterone levels were below the normal range for men and estradiol levels were increased in both postmenopausal women and men [39]. Male sex steroids appear to be immunosuppressive, whereas female sex steroids increase the activity of humoral immune responses. The major source of enhanced estradiol production has been suggested to be the aromatization of testosterone to estradiol [39–41]. The potential role of ADTS/DHTS in sepsis remains to be clarified in future studies.

Another important finding in the present study was that circulating citrulline levels decreased significantly in patients with complicated course of febrile neutropenia. L-Citrulline is used as a biomarker of enterocyte functional mass [42]. Studies in patients with septic shock or those with multiple organ failure have shown that patients with the lowest citrulline nadirs had the highest risk of gastrointestinal tract translocation of bacteria, and plasma citrulline concentration at 24 H ≤ 10 *μ*mol/L was an independent prognostic factor for mortality [43, 44]. Herbers et al. [45] studied citrulline concentrations in neutropenic patients after high-dose melphalan and ASCT. The patients with bacteremia had significantly lower citrulline concentrations on the first day of fever than did those without bacteremia. Our results are in line with these findings: citrulline levels were significantly lower in patients with complicated course of febrile neutropenia. Of note, citrulline serves also as a substrate for nitric oxide production and low levels are associated with acute respiratory distress syndrome (ARDS) [46].

In our study, phospholipid PE(18:0/20:4) increased significantly in patients with complicated course of febrile neutropenia. Also, 12 other molecular features, which fulfilled the second most strict criteria, were all phospholipids. PC is the most abundant and PE the second most abundant phospholipid in mammalian cells [47]. PE and phosphatidylserine (PS) are aminophospholipids, and because of their negative charge, they are localized in the inner leaflet on the plasma membrane. Neutrally charged PC and sphingomyelin localize on the outer leaflet [48, 49]. As a response to stimulus, cytoskeleton is reorganized and the asymmetric distribution of the phospholipid membrane is modified with exposure of PS and PE at the cell surface. Cellular blebbing then occurs, ultimately leading to the release of microparticles (MPs) [50, 51]. MPs originate from several cell types such as leukocytes, erythrocytes, platelets, or endothelial cells. They have been implicated in the pathophysiology of a range of diseases, including infectious diseases [49]. Also, a major role has been suggested for MPs in propagating proinflammatory and proanticoagulant activity in sepsis [50].

PE is abundantly present on the outer surface of mammalian MPs of a variety of human cells, and PE provides a sensitive molecular marker for detecting and characterizing MPs [50]. Clark et al. [48] studied more specifically activated human platelets and found that they externalize two PSs and five PEs: PS(18:0a/20:4), PS(18:0a/18:1), PE(16:0p/20:4), PE(18:0a/18:0), PE(18:1p/20:4), PE(18:0p/20:4), and PE(18:0a/20:4). These are also present in MPs from activated platelets. PS is required for coagulation to take place, and PE can further enhance these reactions. As the acyl-chain length of PE is an essential determinant of this enhancing effect to thrombin generation with PS, the most effective PE was found to be PE(18:0/20:4).

One of the major findings in our study was the positive association of PE(18:0/20:4) with the development of complicated course of neutropenic sepsis. Previously, PE(18:0/20:4) was found to be one of the relevant phospholipids to detect MPs, but the context with clinical data of sepsis has been unsettled. The studies of Larson et al. [51] and Clark et al. [48] were performed *in vitro*, and there are no studies on PE as predictors on sepsis or neutropenic sepsis in humans.

Changes in the plasma phospholipid profile have been found in sepsis. Previously, Rival et al. [52] observed lower total concentrations of phospholipids in septic patients compared to the reference values. This decrease concerned mainly n-6 and n-3 polyunsaturated fatty acids, especially those with a carbon number ≥ 20, which was associated with mortality. Also in patients with ARDS, a significant decrease in the levels of various fatty acids especially in the polysaturated fatty acids (PUFA) including docosahexaenoic acid (DHA, 22:6n3) has been found. DHA levels were decreased also in patients who were at risk of ARDS [53]. In the experimental study, DHA and eicosapentaenoic acid (EPA, 20:5n3) decreased lipopolysaccharide-induced nuclear factor-kappaB activation and monocyte chemoattractant protein-1 production and increased peroxisome proliferator-activated receptor mRNA expression [54]. The decrease in PUFAs has been assumed to be due to their degeneration by reactive oxygen species or a higher synthesis of inflammatory lipid mediators because these PUFAs are the precursors of eicosanoids and docosanoids, which are involved in inflammation, vasomotricity, and capillary permeability [52].

## 5. Conclusion

Although nontargeted metabolite profiling is a hypothesis-generating method, these results already provide a new insight into metabolite markers to differentiate between noncomplicated and complicated courses of febrile neutropenia. Our study represents the largest study in this patient population, even though the relatively low number of patients reduces the statistical power of the results.

Our findings are consistent with previously published data about citrulline and phospholipids. The current findings are clinically relevant when compared with CRP and PCT, which are widely used biomarkers in febrile neutropenia. Most of the previous studies are performed in animals or *in vitro* in contrast to our prospective clinical study, which highlights the importance of current findings.

The pathways of androgen metabolism merit further studies in patients with febrile neutropenia. The results are preliminary and suggest metabolic changes in patients with complicated febrile neutropenia. Further targeted tests to identify and confirm these changes are needed. The whole pathway of metabolites of steroid synthesis in the adrenal gland should be quantified. If specific, consistent metabolite changes can be identified, a routine laboratory-compatible method may be developed.

## Figures and Tables

**Table 1:** Characteristics for the tentatively identified differential compounds in liquid chromatography-mass spectrometry analysis.

Column	ESI	MW	RT (min)	Tentative identification	ID	Identification based on MS/MS	ANOVA		Groupwise *t*-test Corrected *p* value		Groupwise *t*-test noncorrected *p* value					
							D0	D1					Fold change			
							Corrected	*p* value	D0	D1	D0	D1	D0	D1	VIP D0	VIP D1
RP	−	370.1815	8.957	ADTS or DHTS	MID3559	20ev: 369.1746 [M−H]−; 369.1747 (100), 96.9591 (27), 370.1794 (7), 209.6079 (7)	1.08*E* − 07	8.49*E* − 11	6.80*E* − 04	0.030864	5.33*E* − 07	1.94*E* − 04	−2.37	−1.93	2.65	1.74
HILIC	+	175.0954	6.357	Citrulline	MID16	Verified with standard 10ev: 176.1043 [M+H]+; 159.0762 (100), 70.0653 (68), 113.0692 (46), 176.1043 (15)	1.17*E* − 04	1.05*E* − 06	0.77889	0.042103	0.025201	2.02*E* − 04	1.327	1.572	1.67	1.69
RP	−	767.5465	12.93	PE(18:0/20:4)	LMGP0201	20ev: 766.5635 [M−H]−; 766.5635 (100), 303.2348 (39), 767.5412 (34), 283.2612 (13), 59.0151 (7), 10ev: 768.5594 [M+H]+; 768.5635 (100), 627.5359 (32)	1.43*E* − 06	2.25*E* − 09	0.24644	0.016158	0.002157	4.34*E* − 05	−1.42	−1.63	1.71	1.98
RP	−	837.5525	11.98	PC(15:0/22:6) [M+FA]	LMGP0101	40ev: 836.546 [M+FA−H]−; 327.2326 (100), 241.2187 (42), 283.242 (39), 776.5189 (29), 168.0414 (27), 20ev: 792.5457 [M+H]+; 184.0729 (100), 792.5537 (81), 86.0693 (3)	0.007421	2.95*E* − 04	0.325565	0.199228	0.003948	0.016788	1.225	1.209	1.56	1.56
RP	−	827.5697	12.3	PC(16:0/20:4) [M+FA]	LMGP0101	20ev: 826.5624 [M+FA−H]−; 766.5390 (100), 767.5404 (34), 826.5603 (25)	0.007421	3.00*E* − 04	0.366474	0.180537	0.010339	0.007452	−1.09	−1.09	1.58	1.58
HILIC	+	763.5173	0.706	PE(16:0/22:6)	LMGP0201	20ev: 762.5062 [M−H]−; 327.2371 (100), 762.5071 (72), 344.5837 (55), 255.2369 (38), 764.5264 [M+H]+; 764.5264 (100), 623.4989 (50)	0.024721	0.002518	0.663723	0.142026	0.003572	0.007017	−1.49	−1.44	2.18	1.42
RP	+	805.5637	12.22	PC(16:0/22:6)	LMGP0101	20ev: 806.5696 [M+H]+; 184.0729 (100), 806.5701 (86), 524.3132 (5), 20ev: 850.5625 [M+FA−H]−; 93 (100), 850.5600 (39), 791.5447 (39), 327.2323 (18), 44.9986 (14), 851.5603 (13), 255.2322 (6)	0.027808	0.001026	0.599027	0.187622	0.116353	0.004307	1.099	1.163	1.37	1.71
HILIC	+	809.5935	0.632	PC(18:0/20:4)	LMGP0101	20ev: 854.588 [M+FA−H]−; 794.5697 (100), 854.5934 (29), 795.5785 (21), 303.2318 (11), 283.26 (7); 10ev: 810.6012 [M+H]+; 810.6045 (100), 184.073 (17)	0.09676	0.028541	0.784922	0.504751	0.047911	0.210616	−1.1	−1.06	1.59	0.99
HILIC	+	523.3635	1.08	LysoPC(0:0/18:0)	LMGP0105	40ev: 568.3607 [M+FA−H]−; 508.3418 (100), 283.2632 (30), 224.0657 (10), 44.9993 (9), 20ev: 524.3706 [M+H]+; 184.0731 (100), 524.3689 (10)	0.047961	0.008856	0.84261	0.146463	0.279208	0.014867	1.137	1.354	1.48	1.60
HILIC	+	521.3482	1.091	LysoPC(0:0/18:1)	LMGP0105	20ev: 522.3570 [M+H]+; 184.0738 (100), 522.3606 (7), 86.0964 (3)	0.040335	0.005835	0.84261	0.15821	0.230192	0.017452	1.157	1.355	1.47	1.66
HILIC	+	495.334	1.15	LysoPC(0:0/16:0)	LMGP0105	20ev: 540.3303 [M+FA−H]−; 255.2323 (100), 480.3042 (62), 242.0762 (7); 20ev: 496.3492 [M + H]+; 184.0764 (100), 496.3477 (5), 86.0974 (2)	0.047031	0.007895	0.887095	0.142026	0.436041	0.01111	1.085	1.318	1.47	1.52
HILIC	+	523.3653	1.2	LysoPC(18:0/0:0)	LMGP01050026		0.037508	0.005217	0.84261	0.142026	0.289454	0.008929	1.129	1.365	1.42	1.54
HILIC	+	521.3502	1.215	LysoPC(18:1/0:0)	LMGP0105	40ev: 566.345 [M+FA−H]−; 281.2481 (100), 224.0735 (14), 44.999 (9)	0.024721	0.002743	0.84261	0.146463	0.24612	0.014391	1.159	1.392	1.34	1.54
HILIC	+	519.3347	1.227	LysoPC(18:2/0:0)	LMGP0105	20ev: 520.3400 [M+H]+; 184.0733 (100), 520.339 (10), 104.1067 (2), 86.095 (2), 20ev: 564.3326 [M+FA−H]−; 504.3113 (100), 279.2337 (68), 44.9995 (10)	0.009186	4.33*E* − 04	0.887095	0.142026	0.45312	0.006973	1.093	1.45	1.29	1.50
HILIC	+	495.3345	1.284	LysoPC(16:0/0:0)	LMGP01050018		0.103087	0.031116	0.909764	0.185013	0.609742	0.02529	1.055	1.276	1.48	1.59
HILIC	−	222.02	1.302	Unknown		10ev: 221.0126; 221.012 (100), 148.9898 (35), 130.9797 (12), 94.9242 (5), 92.9242 (3)	0.016106	9.59*E* − 04	0.585329	0.33396	0.013859	0.057424	−1.16	−1.13	1.64	1.14
RP	+	231.1466	2.065	Butyryl-L-carnitine	MID964	Verified with standard. 10ev: 232.1545 [M+H]+; 232.1549 (100), 85.0276 (95), 173.0738 (16), 60.0784 (3)	0.49639	0.218745	0.46469	0.782517	0.047231	0.482634	−1.39	−1.14	1.57	0.81
RP	−	173.9987	2.408	Unknown		20ev: 172.9929 [M−H]−?; 93.0348 (100), 34.9705 (6)	0.404675	0.116603	0.861434	0.273229	0.594768	0.030835	−1.19	−2.01	1.36	1.82
RP	+	250.1785	5.666	Unknown		10ev: 251.1860; 59.05 (100), 117.0906 (19), 41.0397 (7), 251.1881 (6)	0.009147	1.34*E* − 04	0.824211	0.216978	0.380635	0.008023	−1.02	1.27	0.86	1.55
HILIC	+	155.0695	6.772	L-Histidine	MID21	Verified with standard 10ev: 156.0765 [M+H]+; 110.0712 (100), 156.0758 (30), 95.0609 (11), 83.0602 (5)	0.016822	0.001302	0.670033	0.826355	0.008034	0.614635	1.207	1.035	1.76	0.86
RP	−	396.1973	8.611	Pregnenolone sulfate	MID5740	20ev: 395.1903 [M−H]−; 395.1903 (100), 96.9607 (23), 248.8949 (7), 305.7866 (6)	0.082724	0.010827	0.384093	0.398782	0.015051	0.079233	−1.5	−1.38	1.71	1.12
RP	+	567.3333	10.12	LysoPC(22:6/0:0) (RP a)	LMGP0105	20ev: 612.3301 [M+FA−H]−; 552.3112 (100), 327.2352 (25), 283.243 (8), 44.9985 (4), 20ev: 568.3392 [M+H]+; 104.1063 (100), 184.0726 (98), 568.3392 (86), 86.096 (14)	0.008933	1.03*E* − 04	0.457269	0.105666	0.041413	5.08*E* − 04	1.291	1.498	1.23	1.51
RP	+	505.4133	11.69	Unknown		10ev: 506.4203; 60.0816 (100), 61.083 (4)	0.159382	0.029154	0.314458	0.799741	0.01188	0.505812	1.307	1.071	1.79	0.87
RP	−	849.5526	11.87	PC(16:1/22:6) [M+FA]	LMGP0101	40ev: 848.5490 [M+FA−H]−; 327.2322 (100), 253.2164 (52), 283.2444 (49), 478.2944 (31), 44.9987 (30), 20ev: 804.5531 [M+H]+; 184.0718 (100), 804.5512 (67)	0.03311	0.002648	0.366474	0.309525	0.008646	0.041723	1.309	1.235	1.56	1.32
RP	−	801.5533	12.02	PC(16:1/18:2) [M+FA]	LMGP0101	20ev: 756.5562 [M+H]+; 184.0737 (100), 756.5533 (78), 20ev: 800.5511 [M+FA−H]−; 740.5234 (100), 800.545 (53), 279.2312 (15), 253.2161 (11), 44.9988 (6)	0.035314	0.002909	0.372759	0.312787	0.01227	0.043172	−1.5	−1.44	1.76	1.19
RP	+	415.3572	12.15	Unknown		10ev: 416.3633; 416.3617 (100), 164.1129 (4), 191.1039 (3)	0.067283	0.006009	0.513659	0.333893	0.070642	0.049803	−1.2	−1.2	1.33	1.60
RP	+	831.5781	12.33	PC(18:1/22:6)	LMGP0101	20ev: 832.5857 [M+H]+; 184.0738 (100), 832.585 (97), 40ev: 876.5774 [M+FA−H]−; 327.2333 (100), 281.2478 (67), 283.243 (32), 816.5537 (18), 44.9985 (18)	0.009147	1.40*E* − 04	0.534883	0.105666	0.07969	3.23*E* − 04	1.163	1.333	1.44	1.66
RP	+	771.5775	12.58	Unknown PC		20ev: 772.5868 [M+H]+; 772.587 (100), 184.0736 (94), 86.0975 (5), double peak, no MS/MS from the first one	0.356662	0.11337	0.314458	0.848946	0.011944	0.591385	−1.24	1.046	1.98	0.98
RP	+	759.5784	12.8	PC(16:0/18:1)	LMGP0101	20ev: 804.5795 [M+FA−H]−; 745.5581 (28), 804.5754 (26), 281.2484 (14), 255.2315 (6), 44.9982 (3), 20ev: 760.5859 [M+H]+; 184.0732 (100), 760.5862 (82)	0.015602	4.25*E* − 04	0.298009	0.596521	0.008491	0.213038	−1.15	−1.06	1.58	1.24
RP	+	833.5943	12.81	PC(18:0/22:6)	LMGP0101	20ev:834.6028 [M+H]+; 834.5985 (100), 184.0727 (91), 86.0979 (2), 40ev: 878.5934 [M+FA−H]; 327.2332 (100), 283.2625 (48), 283.2436 (36), 818.5716 (20), 508.3402 (20), 44.9983 (10)	0.045589	0.002876	0.653825	0.216978	0.165991	0.007318	1.125	1.251	1.23	1.51
RP	+	837.6245	13.57	Unknown PC		20ev: 838.6312 [M+H]+; 838.6324 (100), 184.0728 (56), 185.0744 (6)	0.077038	0.007688	0.285943	0.660969	0.007543	0.291104	−1.42	−1.13	1.57	1.11
RP	+	787.6087	13.61	PC(18:0/18:1)	LMGP0101	20ev: 788.6156 [M+H]+; 184.0728 (100), 788.6134 (100), 84.0787 (2); 40ev:832.6118 [M+FA−H]−; 281.2489 (100), 283.2635 (46), 772.5866 (14), 44.9979 (11)	0.091692	0.010657	0.301815	0.839344	0.008949	0.568381	−1.32	−1.06	1.60	0.84

The characteristics include both uncorrected and FDR- (Benjamini-Hochberg false discovery rate-) corrected *p* values on d0 and d1, fold changes, variable influence on projection (VIP) values, and identification references, together with parameters for the LC-MS analysis, including chromatography (column), ionization mode in mass spectrometry (Ioni), molecular weight (MW), retention time (RT), and fragment ions in tandem mass spectrometry (MS/MS fragments). *n* = 65 (20 patients with complicated course of febrile neutropenia (complicated) and 65 patients without complicated course of febrile neutropenia (noncomplicated). ADTS: androsterone sulfate; DHTS: dihydrotestosterone sulfate; PC: phosphatidylcholine; PE: phosphatidylethanolamine; LysoPC: lysophosphatidylcholine. ANOVA (analysis of variance) and groupwise *t*-test (unpaired *t*-test) comparing the fold changes between complicated versus noncomplicated patients. *p* values < 0.05. Fold changes = average fold changes when comparing complicated group against noncomplicated group, with *p* values. Fold changes ≥ ± 1.2 were considered significant. Positive values indicate increased plasma levels in complicated versus noncomplicated patients, whereas negative values indicate decreased plasma levels in complicated versus noncomplicated patients. ID identification of metabolites is based on references: MID refers to METLIN database https://metlin.scripps.edu/index.php, LMGP refers to LIPID MAPS database http://lipidmaps.org/data/structure/index.html, and HMDB refers to Human Metabolome Database http://www.hmdb.ca/.

**Table 2:** Characteristics of the patients with complicated febrile neutropenia.

Gender/age (years)	Blood culture	Time	Characteristics of severe sepsis	qSOFA	ICU	Outcome
M/65	*Pseudomonas aeruginosa*	1	Pneumonia, respiratory failure	3	Yes	Died
F/70	Negative, RSV-pneumonia	6	Pneumonia, septic shock	2	Yes	Died
M/47	*Enterococcus faecium*	4	Pneumonia, respiratory failure	1	Yes	Died
M/67	Negative	7	Pneumonia, respiratory failure	2	Yes	Recovered
M/58	*Staphylococcus epidermidis*	2	Septic shock, respiratory failure	2	Yes	Recovered
M/63	*Enterococcus faecium*	5	Respiratory failure, ileus	2	Yes	Recovered
M/68	*Streptococcus mitis*	11	Respiratory failure (need for NIV)	2	No (CCU)	Recovered
M/51	*Enterococcus faecium*	1	Low blood pressure, need for plasma expander	1	No	Recovered
F/65	*Streptococcus mitis*	—	—	—	No	Recovered
M/41	*Streptococcus salivarius*	—	—	—		
M/31	*Gemella morbillorum*	—	—	—	No	Recovered
M/62	*Staphylococcus haemolyticus*	—	—	—	No	Recovered
M/36	*Staphylococcus haemolyticus*	—	—	—	No	Recovered
F/62	*Staphylococcus epidermidis*	—	—	—	No	Recovered
M/65	*Staphylococcus epidermidis*	—	—	—	No	Recovered
M/69	*Enterococcus faecium*	—	—	—	No	Recovered
M/58	*Enterococcus faecium*	—	—	—	No	Recovered
F/49	*Klebsiella oxytoca*	—	—	—	No	Recovered
F/56	*Escherichia coli*	—	—	—	No	Recovered
M/61	*Candida krusei*	—	—	—	No	Recovered

M: male; F: female; Time: days from the onset of fever to severe sepsis; qSOFA: quick sepsis-related organ failure assessment; ICU: intensive care unit; NIV: noninvasive ventilation; CCU: cardiac care unit.

**Table 3:** Influence of gender to androsterone sulfate (ADTS)/dihydrotestosterone sulfate (DHTS).

	Women	Men	*p* values
ADTS/DHTS d0	3.0 × 10^4^ (2.0–5.5 × 10^4^)	6.7 × 10^4^ (4.2–10.7 × 10^4^)	<0.001
ADTS/DHTS d1	3.0 × 10^4^ (2.5–4.2 × 10^4^)	7.0 × 10^4^ (4.1–12 × 10^4^)	<0.001

Medians (IQ). Data are expressed as medians (interquartile ranges).

**Table 4:** Influence of complicated course of fever to androsterone sulfate (ADTS)/dihydrotestosterone sulfate (DHTS).

	Men with complication	Men without complication	*p* values
ADTS/DHTS d0	12.6 × 10^4^ (7.9–14 × 10^4^)	4.9 × 10^4^ (3.6-7.8 × 10^4^)	<0.001
ADTS/DHTS d1	12.6 × 10^4^ (8.3–14 × 10^4^)	5.7 × 10^4^ (3.6–8.8 × 10^4^)	0.001
	Women with complication	Women without complication	
ADTS/DHTS d0	6.8 × 10^4^ (4.2–6.8 × 10^4^)	2.5 × 10^4^ (2.0–4.3 × 10^4^)	0.045
ADTS/DHTS d1	7.0 × 10^4^ (1.9–13 × 10^4^)	2.9 × 10^4^ (2.5–4.0 × 10^4^)	0.322

Data are expressed as medians (interquartile ranges).

**Table 5:** Influence of age to androsterone sulfate (ADTS)/dihydrotestosterone sulfate (DHTS).

	Men under 50 years	Men over 50 years	*p* values
ADTS/DHTS d0	8.5 × 10^4^ (5.6–13 × 10^4^)	5.9 × 10^4^ (3.7–9.3 × 10^4^)	0.085
ADTS/DHTS d1	10 × 10^4^ (6.2–12 × 10^4^)	6.4 × 10^4^ (3.6–10.2 × 10^4^)	0.121
	Women under 50 years	Women over 50 years	
ADTS/DHTS d0	5.9 × 10^4^ (3.5–13.7 × 10^4^)	2.5 × 10^4^ (2.0–4.0 × 10^4^)	0.068
ADTS/DHTS d1	8.5 × 10^4^ (2.8–14.5 × 10^4^)	2.9 × 10^4^ (2.4–4.1 × 10^4^)	0.144

Data are expressed as medians (interquartile ranges).

## Supplementary Materials

Supplementary Materials Supplementary Figure: principal component analysis of the molecular features collected at four analytical modes of liquid chromatography-mass spectrometry analysis.
