# Supplementary material for: Novel Biomarker Candidates for Febrile Neutropenia in Hematological Patients Using Nontargeted Metabolomics
Source: Dis Markers. 2018 Apr 12;2018:6964529. doi: 10.1155/2018/6964529 (PMC5925027; doi:10.1155/2018/6964529)
Supplement: Supplementary Materials — Supplementary Figure: principal component analysis of the molecular features collected at four analytical modes of liquid chromatography-mass spectrometry analysis. [file 6964529.f1.pdf]

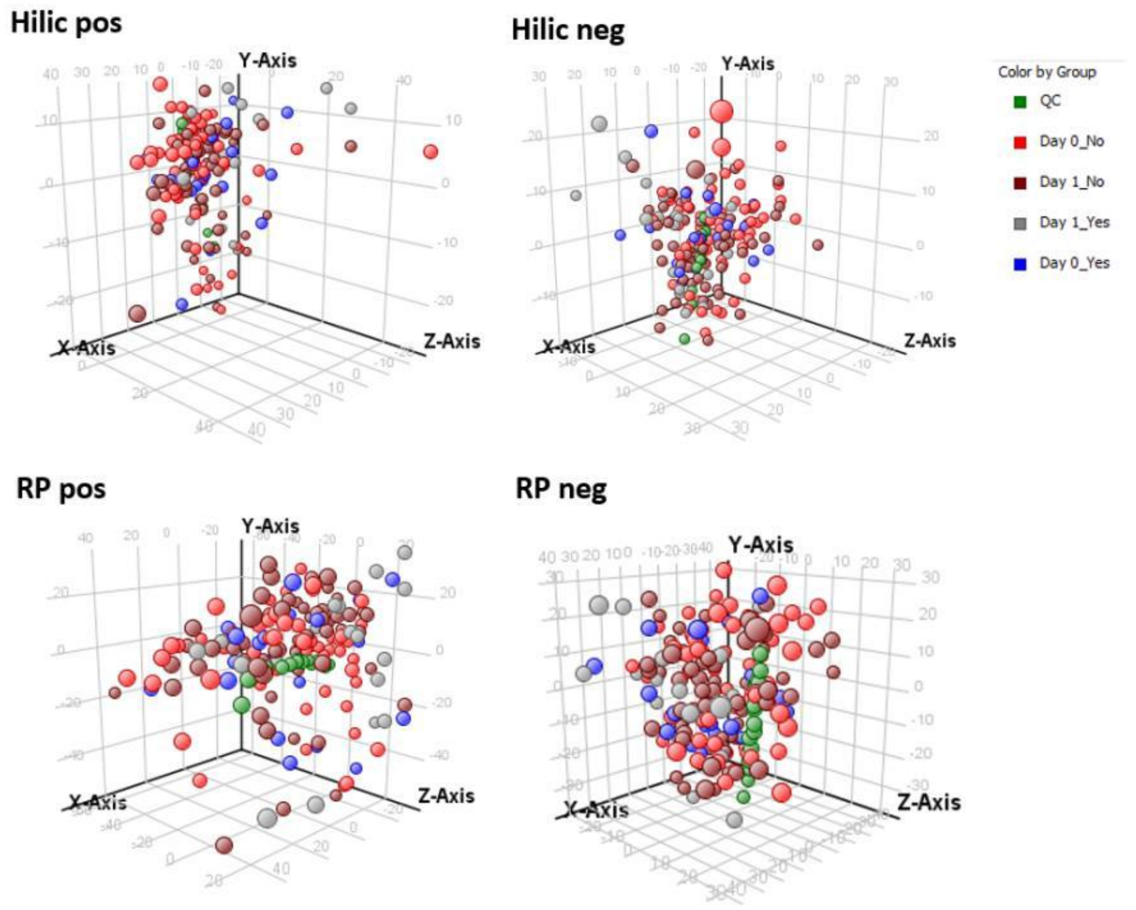

Supplementary Figure. Principal component analysis of the molecular features collected at four analytical modes of liquid chromatography - mass spectrometry analysis. Day 0 and Day 1 refer to the sampling date after the onset of febrile neutropenia and No/Yes refer to the development of complications during febrile neutropenia.
